# Supplementary material for: MetaRibo-Seq measures translation in microbiomes
Source: Nat Commun. 2020 Jun 29;11:3268. doi: 10.1038/s41467-020-17081-z (PMC7324362; doi:10.1038/s41467-020-17081-z)
Supplement: Supplementary file 10 — Supplementary Data 7 [file 41467_2020_17081_MOESM10_ESM.zip › File2/Confidence_High_Taxonomy/259735_out.krona.html]

Javascript must be enabled to view this page.

members
magnitude
magnitudeUnassigned
count
unassigned
taxon
rank

259735\_out

358

358
2
superkingdom

2
976
phylum

2
class
200643

order
171549
2

family
815
2

2
816
genus

1

SRS017701\_contig\_number\_18146
28116
species

1

SRS893285\_contig\_number\_591
371601
species

phylum
1239
3
356

SRS024140\_contig\_number\_contig-100\_16324.16324SRS146764\_contig\_number\_23182SRS147380\_contig\_number\_contig-100\_30160.30161

class
526524
1

1
order
526525

family
128827
1

genus
135858
1

1

SRS019068\_contig\_number\_110118
1946309
species

186801
class
350

order
28
186802

SRS011134\_contig\_number\_contig-100\_3507.309344SRS013965\_contig\_number\_42302SRS015431\_contig\_number\_contig-100\_8495.407979SRS016267\_contig\_number\_9073SRS016753\_contig\_number\_contig-100\_10997.42154SRS016989\_contig\_number\_contig-100\_3900.40422SRS017247\_contig\_number\_176SRS017433\_contig\_number\_contig-100\_8002.46396SRS021219\_contig\_number\_contig-100\_6807.61773SRS023526\_contig\_number\_21133SRS024075\_contig\_number\_16871SRS046369\_contig\_number\_5237SRS049446\_contig\_number\_13839SRS065176\_contig\_number\_contig-100\_4215.88883SRS074970\_contig\_number\_contig-100\_4308.4308SRS077127\_contig\_number\_contig-100\_1100.77052SRS077294\_contig\_number\_contig-100\_182.182SRS098644\_contig\_number\_23177SRS1041039\_contig\_number\_contig-100\_1585.162751SRS1041112\_contig\_number\_4100SRS1041116\_contig\_number\_contig-100\_1675.1676SRS1041130\_contig\_number\_contig-100\_23108.23109SRS1041164\_contig\_number\_contig-100\_5699.5700SRS1055076\_contig\_number\_11326SRS1055095\_contig\_number\_contig-100\_6902.6903SRS143780\_contig\_number\_31721SRS144362\_contig\_number\_contig-100\_642.260292SRS147346\_contig\_number\_61859
350


SRS146813\_contig\_number\_28500
1
species
1897042

2
family
186804

species
1321783

SRS019071\_contig\_number\_21080SRS049253\_contig\_number\_26030
2

186806
family
8

1730
1
8

SRS022524\_contig\_number\_7920
genus

2

SRS048164\_contig\_number\_23891SRS1041037\_contig\_number\_contig-100\_452.94541
165185
species

species
39496

SRS049712\_contig\_number\_18326
1

4

SRS013687\_contig\_number\_16911SRS019601\_contig\_number\_1139SRS056273\_contig\_number\_34566SRS056519\_contig\_number\_19121
2292352
species

1

SRS1041137\_contig\_number\_25663
1950916
species

family

SRS056259\_contig\_number\_contig-100\_32220.67249
44
541000
1

1

SRS017916\_contig\_number\_contig-100\_235.121123
2292270
species

genus
204475
2

species
745368

SRS049995\_contig\_number\_34059SRS098881\_contig\_number\_contig-100\_231.178806
2

species
1898205

SRS016335\_contig\_number\_contig-100\_192.259259SRS050026\_contig\_number\_7544SRS053356\_contig\_number\_45050SRS077502\_contig\_number\_19320SRS097958\_contig\_number\_contig-100\_783.99431SRS101433\_contig\_number\_15976SRS1055049\_contig\_number\_14722
7

genus
292632
1

1

SRS104636\_contig\_number\_17865
2302960
species

genus
216851
1

species
853

SRS893230\_contig\_number\_12540
1

1
1263
31

SRS076976\_contig\_number\_1479
genus

1262950
species
1

SRS016495\_contig\_number\_12179

40518
species
1

SRS019693\_contig\_number\_contig-100\_1713.73634


SRS042628\_contig\_number\_33152
1
species
2293234


SRS014855\_contig\_number\_8087SRS019808\_contig\_number\_19475SRS893366\_contig\_number\_contig-100\_704.156155
3
species
165186


SRS104197\_contig\_number\_452
1
species
41978

1

SRS104400\_contig\_number\_51896
1161942
species

2293148
species
1

SRS148721\_contig\_number\_35851


SRS015578\_contig\_number\_contig-100\_1385.204321SRS015782\_contig\_number\_40219SRS017307\_contig\_number\_contig-100\_458.181998SRS017521\_contig\_number\_51332SRS018541\_contig\_number\_4660SRS019030\_contig\_number\_contig-100\_5.124501SRS022071\_contig\_number\_39244SRS047044\_contig\_number\_19397SRS051610\_contig\_number\_12008SRS062701\_contig\_number\_7904SRS076929\_contig\_number\_27405SRS077641\_contig\_number\_23862SRS097920\_contig\_number\_31960SRS1041090\_contig\_number\_contig-100\_4621.4622SRS1054691\_contig\_number\_10142SRS1055034\_contig\_number\_8890SRS143148\_contig\_number\_25681SRS148784\_contig\_number\_29948SRS149784\_contig\_number\_28654SRS893358\_contig\_number\_6094SRS893378\_contig\_number\_23872
21
species
46228

10
186803

SRS013705\_contig\_number\_25494SRS018300\_contig\_number\_contig-100\_137.112405.112405SRS019178\_contig\_number\_3317SRS023971\_contig\_number\_contig-100\_3223.33257SRS043768\_contig\_number\_29767SRS047433\_contig\_number\_15922SRS098073\_contig\_number\_12220SRS144537\_contig\_number\_contig-100\_2114.252436SRS144753\_contig\_number\_11622SRS150029\_contig\_number\_835
227
family

11
841
45

SRS015217\_contig\_number\_31188SRS015264\_contig\_number\_23064SRS055982\_contig\_number\_14005SRS064276\_contig\_number\_43212SRS077730\_contig\_number\_10305SRS1041033\_contig\_number\_30092SRS1041133\_contig\_number\_25920SRS1041136\_contig\_number\_contig-100\_771.146495SRS104311\_contig\_number\_41652SRS1055022\_contig\_number\_25733SRS143417\_contig\_number\_contig-100\_399.192058
genus


SRS013158\_contig\_number\_5548SRS014613\_contig\_number\_22611SRS015579\_contig\_number\_27446SRS018623\_contig\_number\_18354SRS019910\_contig\_number\_8587SRS021948\_contig\_number\_7260SRS045713\_contig\_number\_19303SRS045739\_contig\_number\_7792SRS046068\_contig\_number\_contig-100\_384.385SRS047014\_contig\_number\_37590SRS049900\_contig\_number\_16257SRS054956\_contig\_number\_14847SRS058070\_contig\_number\_23253SRS063040\_contig\_number\_21310SRS063370\_contig\_number\_contig-100\_4478.61832SRS074670\_contig\_number\_5414SRS075773\_contig\_number\_39256SRS076756\_contig\_number\_15871SRS098061\_contig\_number\_14189SRS098827\_contig\_number\_13717SRS1041038\_contig\_number\_9897SRS104485\_contig\_number\_contig-100\_2196.46001SRS142503\_contig\_number\_57497SRS147919\_contig\_number\_20116SRS148319\_contig\_number\_8462SRS149075\_contig\_number\_contig-100\_1914.116975SRS893369\_contig\_number\_contig-100\_1291.73123
27
species
166486

1

SRS1041132\_contig\_number\_3295
301301
species

3

SRS013476\_contig\_number\_42338SRS051882\_contig\_number\_22591SRS147557\_contig\_number\_30088
360807
species

2292367
species
2

SRS050422\_contig\_number\_33833SRS1055069\_contig\_number\_contig-100\_635.96232

2293138
species
1

SRS098655\_contig\_number\_11231

89

SRS014736\_contig\_number\_contig-100\_2685.65382SRS015065\_contig\_number\_37538SRS018656\_contig\_number\_contig-100\_620.79712SRS018817\_contig\_number\_contig-100\_646.147167SRS018888\_contig\_number\_5013SRS019161\_contig\_number\_42635SRS020233\_contig\_number\_contig-100\_1632.343979SRS020869\_contig\_number\_39292SRS023914\_contig\_number\_29380SRS024132\_contig\_number\_30909SRS024388\_contig\_number\_contig-100\_1933.62648SRS049773\_contig\_number\_35960SRS065504\_contig\_number\_34725SRS074964\_contig\_number\_contig-100\_1248.58892SRS075716\_contig\_number\_2175SRS077194\_contig\_number\_4852SRS077231\_contig\_number\_contig-100\_1161.156701SRS077552\_contig\_number\_contig-100\_1146.168446SRS078419\_contig\_number\_37679SRS097889\_contig\_number\_contig-100\_414.228068SRS1041091\_contig\_number\_22843SRS105153\_contig\_number\_36913SRS143372\_contig\_number\_contig-100\_1863.86546SRS144135\_contig\_number\_32265SRS146812\_contig\_number\_43659SRS148159\_contig\_number\_16398SRS148817\_contig\_number\_14259SRS892882\_contig\_number\_8676
572511
28
genus

33038
species
24

SRS013638\_contig\_number\_9479SRS015854\_contig\_number\_442SRS016954\_contig\_number\_contig-100\_116.159812SRS022137\_contig\_number\_22162SRS024331\_contig\_number\_37905SRS024492\_contig\_number\_20190SRS043001\_contig\_number\_11106SRS045004\_contig\_number\_24029SRS048870\_contig\_number\_22895SRS054905\_contig\_number\_23723SRS055017\_contig\_number\_20087SRS077392\_contig\_number\_contig-100\_919.257011SRS1041146\_contig\_number\_contig-100\_2062.2063SRS104975\_contig\_number\_19881SRS1055043\_contig\_number\_27666SRS143523\_contig\_number\_10924SRS143598\_contig\_number\_contig-100\_956.141221SRS144183\_contig\_number\_38814SRS148091\_contig\_number\_11737SRS148424\_contig\_number\_36318SRS149879\_contig\_number\_24126SRS893170\_contig\_number\_12171SRS893279\_contig\_number\_contig-100\_1780.103835SRS971275\_contig\_number\_contig-100\_1328.274128


SRS011405\_contig\_number\_11481SRS013951\_contig\_number\_24294SRS014287\_contig\_number\_10651SRS014313\_contig\_number\_1958SRS016203\_contig\_number\_contig-100\_274.82627SRS020394\_contig\_number\_contig-100\_116.114580SRS024625\_contig\_number\_contig-100\_183.119998SRS062654\_contig\_number\_26824SRS893172\_contig\_number\_10080SRS893373\_contig\_number\_contig-100\_339.156710
10
species
40520

1

SRS019445\_contig\_number\_31350
2292969
species

3

SRS015431\_contig\_number\_32253SRS046717\_contig\_number\_8932SRS075341\_contig\_number\_26107
33035
species

species
33039

SRS014923\_contig\_number\_42787SRS015190\_contig\_number\_16543SRS019685\_contig\_number\_38676SRS043411\_contig\_number\_contig-100\_543.109455SRS056259\_contig\_number\_31388SRS063190\_contig\_number\_15270SRS063518\_contig\_number\_39795SRS077454\_contig\_number\_contig-100\_491.120264SRS104036\_contig\_number\_7987SRS1041031\_contig\_number\_30379SRS1041032\_contig\_number\_16170SRS1041134\_contig\_number\_5819SRS1055038\_contig\_number\_6555SRS143342\_contig\_number\_15319SRS143876\_contig\_number\_contig-100\_148.236531SRS144506\_contig\_number\_57816SRS147377\_contig\_number\_8668SRS148721\_contig\_number\_47351SRS893252\_contig\_number\_contig-100\_908.118561SRS971276\_contig\_number\_28474
20


SRS015694\_contig\_number\_20689SRS142781\_contig\_number\_11454SRS147977\_contig\_number\_13247
3
species
1520805


SRS018313\_contig\_number\_6981
1
species
1952137

genus
830
1

species
1520809

SRS144014\_contig\_number\_25150
1

5

SRS012969\_contig\_number\_40061SRS063813\_contig\_number\_3947SRS075984\_contig\_number\_26698SRS147022\_contig\_number\_11845SRS893231\_contig\_number\_2272
39491
species

8
genus
2005359

39482
species
8

SRS014235\_contig\_number\_42711SRS022609\_contig\_number\_48974SRS023715\_contig\_number\_contig-100\_929.176704SRS043701\_contig\_number\_12402SRS098644\_contig\_number\_35208SRS142599\_contig\_number\_32088SRS147139\_contig\_number\_45292SRS893187\_contig\_number\_11231

1506553
genus
19

species
1531

SRS016056\_contig\_number\_contig-100\_2894.39654SRS017103\_contig\_number\_27244SRS017191\_contig\_number\_19088SRS017821\_contig\_number\_33879SRS018575\_contig\_number\_8491SRS019968\_contig\_number\_464SRS023583\_contig\_number\_contig-100\_63.95577SRS048060\_contig\_number\_contig-100\_1988.85550SRS049896\_contig\_number\_30210SRS050752\_contig\_number\_contig-100\_2279.182958SRS063985\_contig\_number\_32419SRS065397\_contig\_number\_15245SRS098571\_contig\_number\_62524SRS098717\_contig\_number\_23813SRS104084\_contig\_number\_contig-100\_109.90435SRS1041141\_contig\_number\_4609SRS142890\_contig\_number\_contig-100\_2560.246401SRS142923\_contig\_number\_contig-100\_93.132235
18

species
208479

SRS018936\_contig\_number\_760
1

genus
207244
7


SRS011084\_contig\_number\_33267SRS013800\_contig\_number\_contig-100\_24.160255SRS021484\_contig\_number\_32417SRS024435\_contig\_number\_41098SRS1041147\_contig\_number\_668SRS143991\_contig\_number\_17455SRS149244\_contig\_number\_13143
7
species
649756

genus
189330
28

3

SRS1055067\_contig\_number\_15945SRS1055099\_contig\_number\_contig-100\_1360.69727SRS140492\_contig\_number\_6837
286138
species

1

SRS014948\_contig\_number\_contig-100\_1094.141388
2293098
species

5

SRS013098\_contig\_number\_38178SRS1041092\_contig\_number\_contig-100\_1527.90185SRS1054716\_contig\_number\_9778SRS149181\_contig\_number\_32021SRS893270\_contig\_number\_25428
39486
species

88431
species
6

SRS016095\_contig\_number\_14064SRS018836\_contig\_number\_27008SRS045645\_contig\_number\_4596SRS049318\_contig\_number\_33905SRS075021\_contig\_number\_6579SRS148196\_contig\_number\_41630

2293099
species
13

SRS014459\_contig\_number\_42353SRS018936\_contig\_number\_4795SRS019381\_contig\_number\_22117SRS050299\_contig\_number\_12478SRS052027\_contig\_number\_20699SRS064557\_contig\_number\_contig-100\_173.126532SRS077086\_contig\_number\_14401SRS1041140\_contig\_number\_14707SRS142542\_contig\_number\_contig-100\_517.166159SRS143466\_contig\_number\_30488SRS147425\_contig\_number\_11647SRS149325\_contig\_number\_17155SRS893380\_contig\_number\_1445

33042
genus
14

3

SRS050998\_contig\_number\_14939SRS893259\_contig\_number\_712SRS893288\_contig\_number\_contig-100\_119.43249
410072
species

2

SRS104912\_contig\_number\_3515SRS148874\_contig\_number\_14796
458253
species

9

SRS015663\_contig\_number\_37071SRS019496\_contig\_number\_contig-100\_386.113276SRS048981\_contig\_number\_8031SRS049402\_contig\_number\_14908SRS051031\_contig\_number\_contig-100\_1361.200317SRS053214\_contig\_number\_22658SRS1041138\_contig\_number\_508SRS1041142\_contig\_number\_contig-100\_79.62306SRS140513\_contig\_number\_11087
116085
species


SRS018427\_contig\_number\_30458
1
species
1897043

216572
family
4

genus
459786
4

species
1945593

SRS053649\_contig\_number\_22667SRS063518\_contig\_number\_34766SRS145497\_contig\_number\_18510SRS893295\_contig\_number\_10506
4

species
1497955

SRS146888\_contig\_number\_15373
1

33
31979
family

33

SRS018984\_contig\_number\_33926SRS075078\_contig\_number\_contig-100\_667.202435SRS076804\_contig\_number\_contig-100\_2237.127410
3
1485
genus

species
2292998

SRS012273\_contig\_number\_48439SRS013940\_contig\_number\_contig-100\_1643.141144SRS014979\_contig\_number\_23754SRS045244\_contig\_number\_1460SRS062427\_contig\_number\_3053SRS077024\_contig\_number\_7461SRS1055083\_contig\_number\_12688SRS147614\_contig\_number\_contig-100\_2919.71130
8

species
1262820

SRS011302\_contig\_number\_23444
1

species
1263068

SRS1041129\_contig\_number\_13306
1

species
59620

SRS014683\_contig\_number\_3733SRS015890\_contig\_number\_24124SRS016018\_contig\_number\_9934SRS018351\_contig\_number\_contig-100\_783.117207SRS019397\_contig\_number\_13559SRS049959\_contig\_number\_41061SRS054590\_contig\_number\_contig-100\_328.29337SRS1041095\_contig\_number\_6131SRS142712\_contig\_number\_5715
9

1262781
species
1

SRS147766\_contig\_number\_1424


SRS011239\_contig\_number\_26628
1
species
2293018

species
2293008

SRS893292\_contig\_number\_contig-100\_145.65906SRS893300\_contig\_number\_11573
2

36834
species
1

SRS015960\_contig\_number\_23117


SRS012849\_contig\_number\_contig-100\_2305.213367SRS013215\_contig\_number\_contig-100\_3152.3152SRS017859\_contig\_number\_contig-100\_4760.40798SRS024549\_contig\_number\_contig-100\_1749.47826SRS075821\_contig\_number\_contig-100\_1293.140533SRS893383\_contig\_number\_33959
6
species
2293006

91061
class
2

186826
order
2

2
family
1300

2
1301
genus

species group
671232
2

species
1338

SRS015794\_contig\_number\_10660SRS893342\_contig\_number\_3911
2
